# In-ice light measurements during the MOSAiC expedition

**DOI:** 10.1038/s41597-024-03472-0

**Published:** 2024-06-27

**Authors:** Niels Fuchs, Philipp Anhaus, Mario Hoppmann, Torbjoern Kagel, Christian Katlein, Ronja Reese, Leif Riemenschneider, Ran Tao, Ricarda Winkelmann, Dirk Notz

**Affiliations:** 1 Institute of Oceanography, Center for Earth System Research and Sustainability (CEN), Universität Hamburg, 20146 Hamburg, Germany; 2 Alfred-Wegener-Institut, Helmholtz-Zentrum für Polar- und Meeresforschung, 27570 Bremerhaven, Germany; 3 Potsdam Institute for Climate Impact Research (PIK), Member of the Leibniz Association, 14473 Potsdam, Germany; 4 Department of Geography and Environmental Sciences, Northumbria University, Newcastle, UK; 5 Institute for Materials Science and Max Bergmann Center for Biomaterials, Dresden University of Technology, 01069 Dresden, Germany; 6 FutureLab on Earth Resilience in the Anthropocene, Earth System Analysis, Potsdam Institute for Climate Impact Research (PIK), Member of the Leibniz Association, 14473 Potsdam, Germany

**Keywords:** Cryospheric science, Marine biology, Physical oceanography, Cryospheric science

## Abstract

We present light measurements in Arctic sea ice obtained during the year-long MOSAiC drift through the central Arctic Ocean in 2019–2020. Such measurements are important as sea ice plays a fundamental role in the Arctic climate and ecosystem. The partitioning of solar irradiance determines the availability of radiation energy for thermodynamic processes and primary productivity. However, observations of light partitioning along the vertical path through the ice are rare. The data we present were collected by two measurement systems, the lightharp and the lightchain, both measuring autonomously multi-spectral light intensity in different depths within the ice. We present the dataset, retrieval methods for derived optical properties, and the conversion into the final, freely available data product, following standardized conventions. We particularly focus on the specifications of the newly developed lightharp system. Combined with the interdisciplinary and multi-instrument setup of MOSAiC, we expect great potential of the dataset to foster our understanding of light transmission and reflection in the sea-ice cover and interactions with physical sea-ice properties and the polar ecosystem.

## Background & Summary

The year-round Arctic sea ice cover affects almost all physical and ecological processes in the Arctic Ocean^1^. Many of these processes are strongly interlinked with the partitioning of solar irradiance at the sea ice surface, within the ice and underneath it. This partitioning alters the radiation budget of the atmosphere and upper ocean and governs the availability of photosynthetically active radiation (PAR) for primary productivity^2,3^.

**Fig. 1:**
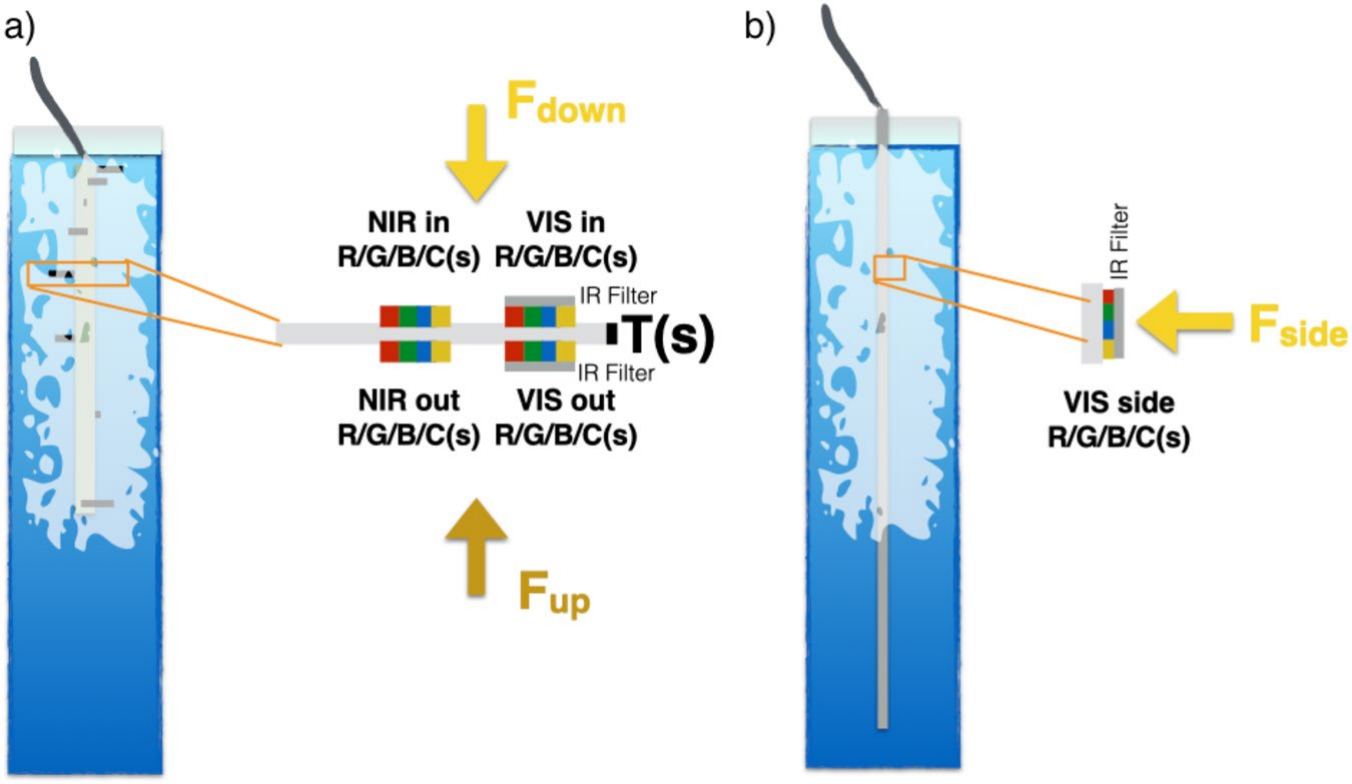
Sketch of the lightharp (**a**) and lightchain (**b**) frozen into the ice, and a close-up of an individual light sensor module. The lightharp consists of 8 such sensor modules (IDs 0 to 7), while the lightchain incorporates 64 sensors (IDs 1 to 64). F indicates planar irradiance in the ice from different directions.

Despite this importance, we lack understanding of the complex interaction between physical sea ice properties, processes that control the optical properties of the ice, their interaction with the ecosystem^4,5^ and their seasonal evolution^6^. To address this gap, during the one-year-long Multidisciplinary drifting Observatory for the Study of Arctic Climate (MOSAiC) drift campaign in the central Arctic Ocean from 2019 to 2020, a comprehensive dataset of the coupled system was collected in an interdisciplinary and integrated approach to advance process understanding and to improve future predictions of the Arctic climate system^7–9^. As part of MOSAiC, physical and biological data from sea ice were collected over the course of roughly one year by *in situ* ice coring and a fleet of autonomous buoys^9^. We here present the data records obtained by two different measurement systems as part of this buoy array designed to detect the light field within the ice: the lightchain sensor and the newly developed lightharp system (see Fig. 1). While a technical description of the former was given in detail by Katlein *et al*.^10^, we here explicitly include the design concept, development, and data processing of the lightharp buoy, which is necessary for the interpretation of the data.

The lightharp buoy has been developed to measure the up- and downwelling irradiance in the ice at 8 different depths. It was deployed during MOSAiC in second-year ice at the so-called *LM site* from January to August 2020. This site was far away from any artificial light sources to allow for undisturbed measurements of sea-ice optics and biology. The aim of the lightharp deployment was to investigate light partitioning, its drivers, and its impacts in conjunction with the other activities at this site, including regular ice coring.

The key design element of the lightharp system is its focus on vertical gradients of up- and downwelling light within the ice and, with that, the ability to measure vertically entering and exiting light from discrete layers. This partitioning allows us to retrieve apparent optical properties (AOPs)^11^, such as transmittance, reflectance, and absorptance in 8 spectral bands, within 7 stacked horizontal ice layers. Furthermore, we derive calibrated PAR in 8 different ice depths, located between the snow–ice interface and 1.4 m depth.

In contrast, lightchains measure the sideward planar irradiance to estimate the scalar irradiance. Measurements are retrieved from 64 different depths at 0.05 m spacing, covering a total length of 3.15 m. Because of their length, the lightchains can measure, for example, the light field from above the surface snow layer to below the ice–ocean interface. Three buoy systems equipped with lightchains were deployed at four locations during MOSAiC (including one redeployment). These buoys are generally referred to as *spectral radiation stations*. They were additionally equipped with 3 RAMSES spectral radiometers (TriOS Optical Sensors, Rastede, Germany) that recorded spectrally resolved incoming, reflected, and downwelling transmitted planar solar irradiance. All three systems were initially deployed on MOSAiC Legs 3–4: one co-located with the lightharp at the above-mentioned *LM site* (2020R11^12^), one at a remote buoy location called the *L3 site* that was part of a distributed network^13^ of autonomous buoys around the MOSAiC sampling site (2020R12^14^), and one in the *Central Observatory* (CO) of the MOSAiC expedition (2020R10^15^). Buoy 2020R11 was recovered in late July/early August 2020 and was redeployed on Leg 5 within a melt pond at the main *buoy site* as 2020R21^16^.

In this study, we present the dataset of in-ice light data collected by these systems during MOSAiC (Table 1). This data, in particular, when combined with textural and inherent optical properties of ice cores and other radiance measurements from MOSAiC, provide a unique data basis to foster radiative transfer process understanding and thereby improve prediction capabilities of light transmittance.

**Table 1:** In-ice light measurement systems included in the data description.

System	Station	MOSAiC site	Ice type	Data period	Raw data	Protocols
Lightharp	—	LM site	SYI	2020-01-15 to 2020-07-18	DOI	Report
Lightchain	2020R10	CO	FYI	2020-03-08 to 2020-03-23	DOI	Report
Lightchain	2020R11	LM site	SYI	2020-03-26 to 2020-07-22	DOI	Report
Lightchain	2020R12	L3 site	FYI	2020-04-24 to 2020-08-07	DOI	Report
Lightchain	2020R21	CO3 pond site	FYI	2020-08-25 to 2020-10-25	DOI	Report

2020R10 was located in the central observatory (CO) between the end of *ROV optics grid* (Transponder 2) and *Fort Ridge*. Ice types include second-year ice (SYI) that formed in 2018–2019 and survived the melting period of summer 2019, and first-year ice (FYI) that formed in winter 2019–2020.

We will first introduce the systems’ design and how optical quantities within sea ice are derived from the obtained data. We will then explain the processing and validation of the MOSAiC measurements, which yield a comprehensive dataset for studies of the physical sea ice system and the ice-associated ecosystem.

## Methods

The datasets presented in this paper were obtained using two different, uniquely designed instruments: the lightharp and lightchain. First, we introduce the technical details of the instruments. Then, we describe in detail the deployment and resulting datasets. The lightchains were already comprehensively described in Katlein *et al*.^10^ and are therefore only briefly summarized here. The lightharp, however, is an entirely new development, and the following paragraphs illustrate this particular system’s specifications, measurement principles, and processing methods.

### Lightharp concept, design and specifications

#### Layer scheme

**Fig. 2:**
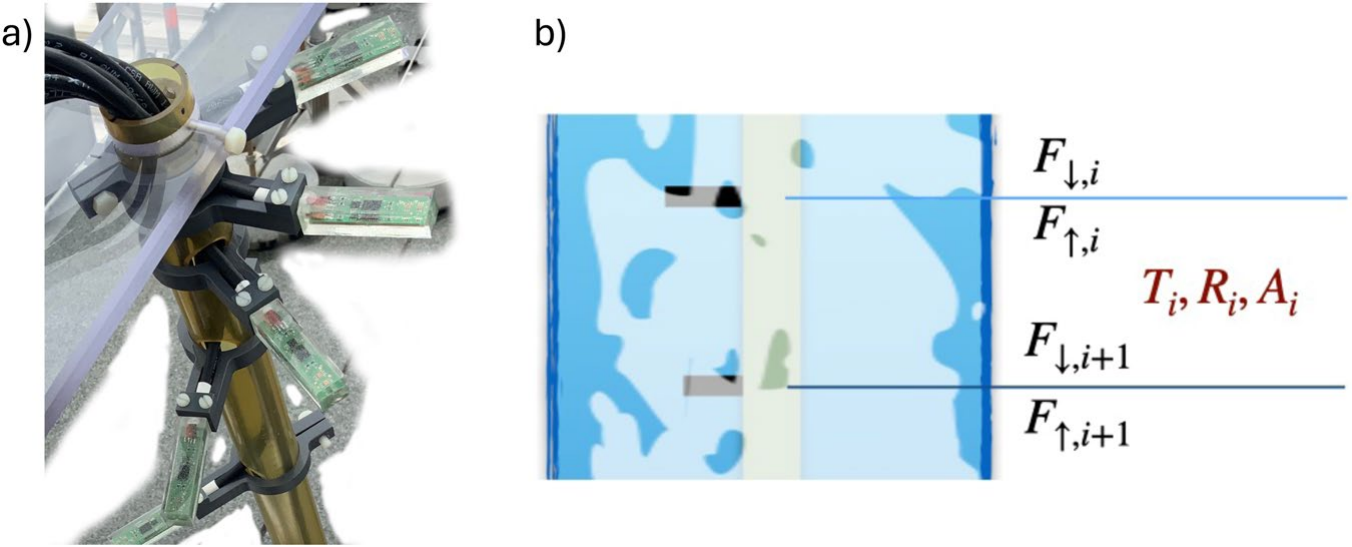
Picture of the upper five sensors of the lightharp (**a**) and sketch showing the layer scheme (**b**). Layers are defined between lightharp sensor module pairs. Derived apparent optical properties are absorptance *A*, transmittance *T* and reflectance *R*.

As the incident light passes through the ice, its intensity decreases from top to bottom due to scattering and absorption^17^. To quantify the apparent optical properties of the ice, we conceptually divide it into horizontal layers and assume these have uniform optical properties for up- and downwelling light (Fig. 2b).

Conceptually speaking, when we neglect horizontal gradients, outgoing light *F*_*out, i*_ from a layer *i* is given as the sum of the downwelling component of the light flux *F*_↓, *i*+1_ at the bottom interface of a layer *i* + 1 and the upwelling light flux *F*_↑, *i*_ at the top interface *i*, while incoming light *F*_*in, i*_ is given by upwelling light at the bottom *F*_↑, *i*+1_ and downwelling light at the top *F*_↓, *i*_. The ratio between incoming and outgoing light yields the apparent optical absorptance *A*_*i*_ of a layer *i*:

1 $${A}_{i}=1-\left({F}_{out,i}/{F}_{in,i}\right).$$

To retrieve the apparent optical reflectance *R*_*i*_ and transmittance *T*_*i*_ of the layer, we equate up- and downwelling light *F*_↑, *i*_, *F*_↓, *i*_ at each layer interface with the sum of back-reflected and transmitted light from the layer above or below, respectively.

2 $${F}_{\downarrow ,i+1}={R}_{i}\cdot {F}_{\uparrow ,i+1}+{T}_{i}\cdot {F}_{\downarrow ,i}$$

3 $${F}_{\uparrow ,i}={R}_{i}\cdot {F}_{\downarrow ,i}+{T}_{i}\cdot {F}_{\uparrow ,i+1}$$

We solve these for *R* and *T* and retrieve a system of 2 equations that yields reflectance and transmittance for each layer *i* from measurements at the upper layer interface *i* and lower interface *i* + 1:

4 $${R}_{i}=\frac{{F}_{\uparrow ,i+1}\cdot {F}_{\downarrow ,i+1}-{F}_{\uparrow ,i}\cdot {F}_{\downarrow ,i}}{{\left({F}_{\uparrow ,i+1}\right)}^{2}-{\left({F}_{\downarrow ,i}\right)}^{2}}$$

5 $${T}_{i}=\frac{{F}_{\uparrow ,i+1}\cdot {F}_{\uparrow ,i}-{F}_{\downarrow ,i+1}\cdot {F}_{\downarrow ,i}}{{\left({F}_{\uparrow ,i+1}\right)}^{2}-{\left({F}_{\downarrow ,i}\right)}^{2}}$$

Equations 1, 4 and 5 together fulfill the law of conservation of energy:

6 $${A}_{i}+{R}_{i}+{T}_{i}=1$$

#### Lightharp design

The lightharp - named in line with the previously developed saltharps^18^ - was developed and built at the Max-Planck Institute for Meteorology in Hamburg/Germany and at Universität Hamburg to obtain internal optical sea-ice properties throughout an entire year, including during freezing and melting. The outlined layer model guided the instrument design. The lightharp is deployed into a hole in the ice at an early stage in the freezing season, freezes in, and then measures autonomously and non-destructively within the ice. The lightharp consists of 8 sensor modules (numbered 0 to 7) that measure up- and downwelling light in 8 different ice depths, yielding information about 7 layers in between and boundary fluxes beyond (see Figs. 1a, 2a). Each module hosts 4 sensors that measure up- and downwelling light in 8 different spectral bands presented in the next section. Modules are mounted perpendicular to a central tube, with a total diameter of less than an 8-inch ice auger borehole width to facilitate the installation and to reduce the impact of deployment on the surrounding ice. The vertical spacing between the modules used during MOSAiC has been logarithmic, with smaller distances towards the top, as stronger vertical gradients are expected there. The sensor depths were from top downwards 0.0 m, 0.015 m, 0.045 m, 0.1 m, 0.21 m, 0.465 m, 0.95 m, and 1.4 m. To minimize optical interference such as shading and reflections, the modules are helically aligned around the central tube; furthermore, all wiring is laid on the inside of the tube, the tube is fabricated in a dark and translucent material, and sensors are located on the module tips, approximately 0.07 m away from the tube.

#### Lightharp sensors

**Fig. 3:**
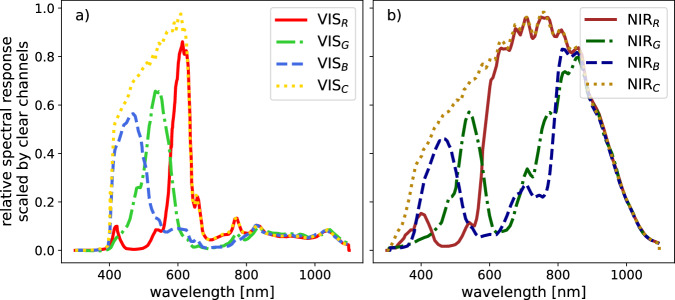
Relative spectral response curves of the VIS (**a**) and NIR (**b**) color bands, given in arbitrary units scaled by the maximum of the clear channels. (Curves reproduced from technical datasheets of the manufacturer^37,38^).

The lightharp sensors, manufactured by *ams Sensors Germany GmbH*, are highly sensitive light-to-digital converters measuring light intensity with red-filtered, green-filtered, blue-filtered, and clear (unfiltered) photodiodes. To broaden the recorded spectrum, two sensors were mounted next to each other: a sensor for the visible spectrum (VIS) with a pre-filter to cutoff near-infrared radiation (TCS3472) and one without (TCS3471) that covers the visible and near-infrared spectrum (NIR) (see Fig. 3). The VIS sensor is identical to the one used in the lightchain system. Both sensor types measure light intensity in three distinct wavelength bands and one clear channel that approximately envelops the spectral range of the other three. The three narrow spectra of the TCS3472 sensor equal the common R, G, and B channels of the RGB color space. Temperature responsiveness is negligible in the VIS spectra with a temperature coefficient below 0.02%K^−1^. In the NIR spectra, temperature responsiveness increases exponentially from 0.03%K^−1^ at 700 to 8%K^−1^ at 1000 nm. Sensors are mounted parallel to the horizontal plane, with two upward oriented sensors *s*_*m, d, VIS/NIR*_ on each module *m* measuring downwelling planar irradiance *E*_*d*_ and two downward oriented sensors *s*_*m, u, VIS/NIR*_ for upwelling planar irradiance *E*_*u*_. Measurements are provided as uncalibrated digital numbers (DN) with 16 bits brightness levels per color band, responding linearly proportional to measured irradiance. The TCS3472 sensor consists of a 3 × 4 photodiode array, averaging across 3 photodiodes per color band (VIS_*R*_, VIS_*G*_, VIS_*B*_, VIS_*C*_), while the TCS3471 consists of a 4 × 4 photodiode array, with 4 photodiodes per color band (NIR_*R*_, NIR_*G*_, NIR_*B*_, NIR_*C*_).

To cover the broad range of light conditions in Arctic sea ice, we made use of a gain factor functionality of the sensors with a customized optimization algorithm that allows for linear scaling of the 16 bits DN with factors 1x, 4x, 16x and 60x. This means that in addition to the entire data range from 0 DN to 65535 DN with resolution 1 DN, a subdivision for small values is available, allowing for the finest resolution of 1/60 DN in the range 0 DN to 1091.3 DN. This extra functionality, while requiring more power and time for a single profile measurement to determine the correct gain, was motivated by our aim to collect photosynthetically active radiation (PAR) at extremely low light levels to allow for a better understanding of the response of the ecosystem to the returning light after polar night.

In addition to light, the lightharp also measures temperature at each module depth with an *Microchip Technology Inc*. MCP9808 temperature sensor that has an accuracy of about ±0.25 °C from −40 °C to 125 °C. Assembled module fingers were molded entirely in transparent epoxy resin to provide the best protection from the harsh environmental conditions.

#### Data acquisition and processing

The lightharp array is connected to a customized, power-effective data acquisition system, installed together with a battery power supply (nominal capacity of ≈7 kWh allowing for approximately two years of multiple measurements per day) in a protective case. This case was placed on the ice some meters away from the optical measurement footprint.

The control unit acquires the intensity measurements from all sensors at a pre-set measurement frequency, stores the data on an SD card, and simultaneously transmits the data via IRIDIUM satellites as short burst data (SBD). In the absence of a dedicated GPS receiver, the satellite link allows for a coarse determination of the unit’s geographic location during data transmission using Doppler calculation, which is a power-effective GNSS solution sufficiently precise in a constantly drifting environment.

A complete measurement cycle through all sensors required 38 min during the MOSAiC deployment. After two consecutive modules were read, their data was collectively transmitted per SBD and stored internally. The maximum transmission quota of SBDs determined the grouping into two modules. Hence, every completed profile eventually consists of 4 subsets to cover all eight modules. Each stored and transmitted data string of a subset contains one UTC timestamp generated from an integrated real-time clock (RTC), the module pair numbers (m1, m2) out of (0, 1),(2, 3),(4, 5),(6, 7), the temperature at the module depth *T*_*m*_, raw measurements of each optical sensor band *I*_*raw*_ of down- (*d*) and upwelling (*u*) light, and the gain factors of the sensors *g*_*s*_ in the following format:

Date [YYMMDDhhmmss], M(m1,m2),

T_*m*1_ [°C], [*I*_*m*1,*d,VIS,raw*_]*c,R,G,B*, g_*m*1,*d,VIS*,_ [*I*_*m*1,*d,NIR,raw*_]*c,R,G,B*, g_*m*1,*d,NIR*,_ [*I*_*m*1,*u,VIS,raw*_]*c,R,G,B*, g, g_*m*1,*u,VIS,*_ [*I*_*m*1,*d,NIR,raw*_]*c,R,G,B*, g_*m*1,*u,NIR*,_

T_*m*2_[°C], [*I*_*m*2,*d,VIS,raw*_]*c,R,G,B*, g_*m*2,*d,VIS*,_ [*I*_*m*2,*d,NIR,raw*_]*c,R,G,B*, g_*m*2,*d,NIR*,_ [*I*_*m*2,*u,VIS,raw*_]*c,R,G,B*, g, g_*m*2,*u,VIS*,_ [*I*_*m*2,*d,NIR,raw*_]*c,R,G,B*, g_*m*2,*u,NIR*_

The raw data are stored permanently in this format, separated into 4 ASCII files containing data from sensor modules 0&1, 2&3, 4&5, and 6&7. The raw MOSAiC timeseries is made accessible on the Data Publisher for Earth & Environmental Science PANGAEA^19^.

In post processing, the raw light intensity readings *I*_*s, b, raw*_ of sensors *s* and different spectral bands *b* were corrected linearly with the gain factor *g*_*s*_ using

7 $${I}_{s,b}=\frac{{I}_{s,b,raw}}{{g}_{s}}$$

### Lightharp calibration

The calibration of the lightharp during MOSAiC was built on two pillars: (I) intercomparison of sensor modules on the assembled lightharp and (II) absolute calibration in the field with RAMSES spectroradiometer data. Owing to logistical challenges, an absolute calibration of all sensor bands of the lightharp under controlled laboratory conditions was unfortunately not possible before deployment. Additionally, again for logistical reasons, the instrument could not be retrieved at the end of the expedition, making a subsequent calibration impossible. We thus used the field data from a co-deployed sensor to achieve an absolute calibration of the VIS bands at least. For future deployments, the calibration should ideally be carried out under controlled illumination conditions between the molding of the sensor modules and the assembly of the final harp. As a reference light source, we recommend, for example, an integrating sphere that provides an isotropic, traceable light field.

#### Correction for differences between sensors

**Fig. 4:**
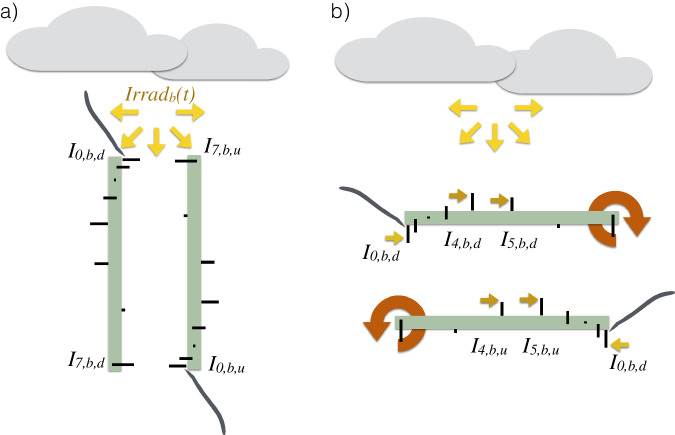
Sketch of the vertical (**a**) and horizontal (**b**) calibration measurements performed with the lightharp under diffuse illumination conditions. The uppermost sensor for downwelling light *I*_0, *b, d*_ for different spectral bands *b* was used as the reference sensor for mutual differences. In the horizontal calibration, the differences between the sensor modules pointing upwards at one point in time were compared and the calibration coefficients were derived continuously from the reference module to the corrected sensor. The temporal evolution of incident light in different spectral bands *Irrad*_*b*_(*t*) was derived from the uppermost sensors.

We tested the lightharp under natural illumination conditions to detect possible deviations between the sensors’ intensity measurements. The conditions were strongly diffuse to reduce misleading directional differences on the helical sensor setup and at twilight. This was necessary to avoid oversaturation of the sensors, whose brightness range was optimized for in-ice measurements. Intercomparison was performed outside on a rooftop of the Alfred Wegener Institute in Bremerhaven, Germany, on 2019-05-08 in the evening hours. Two methods were chosen: a horizontal calibration, in which modules were checked pairwise for differences, and a vertical calibration, in which all sensors were compared simultaneously (Fig. 4). The notation for both methods was chosen to match the orientation of the harp during calibration. For the horizontal calibration, the harp was placed with its central tube horizontally on a structure and rotated successively around the tube so that the tested modules pointed upward into the undisturbed diffuse light field. The harp was then turned so that the sensors for downwelling light could be compared with those for upwelling. Based on this data set, we calculated correction factors consecutively for all sensors and all spectral bands so that they match the measurements of the uppermost sensor pair *s*_0*,d,VIS*_ and *s*_0*,d,NIR*_ for downwelling light that serves as a reference. We assume that the sensors differ only in their linear response to light intensity and the optical thickness of the resin. Therefore, a simple constant correction factor *γ*_*s,b,d/u*_ per sensor *s* and band *b* is sufficient to obtain, separated by orientation downwelling *d* or upwelling *u*,

8 $$\overline{{I}_{0,b,d}}={\gamma }_{s,b,d}\cdot {\overline{{I}_{s,b,d}}}_{,}\quad \quad \quad \overline{{I}_{0,b,d}}={\gamma }_{s,b,u}\cdot \overline{{I}_{s,b,u}}.$$

**Fig. 5:**
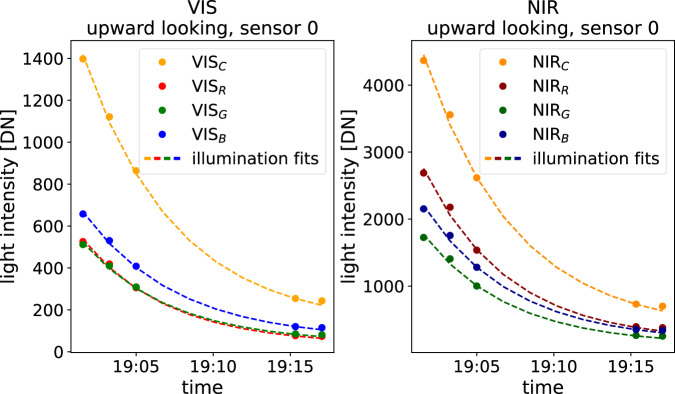
Approximated illumination during the vertical calibration on 2019-05-08 and intensity measurements of the uppermost, upward-orientated sensor 0.

For the second calibration measurement, we mounted the lightharp vertically on the structure. We aimed for a simultaneous incidence of light on one side of all sensors as constant and equal as possible. There may have been a slight shading on the lower sensors, mutually and through the center tube. Cables were placed carefully away from the sensors. As illumination declined fast during this measurement, we de-trended the data with an exponential light decline function *Irrad*_*b*_(*t*), again fitted to the measurements of the uppermost sensor pair for incoming light *s*_0*,d,VIS*_ and *s*_0*,d,NIR*_ at times *t* (shown in Fig. 5). In this case, correction factors *γ*_*s,b,u/d*_ were determined for all sensors relative to this light decline function, such that

9 $$Irra{d}_{b}(t)={\gamma }_{s,b,d}\cdot \overline{{I}_{s,b,d}(t)},\quad \quad \quad Irra{d}_{b}(t)={\gamma }_{s,b,u}\cdot \overline{{I}_{s,b,u}(t)}.$$

For the downward-oriented sensors (index *u*), the harp was flipped, and the procedure was repeated.

**Fig. 6:**
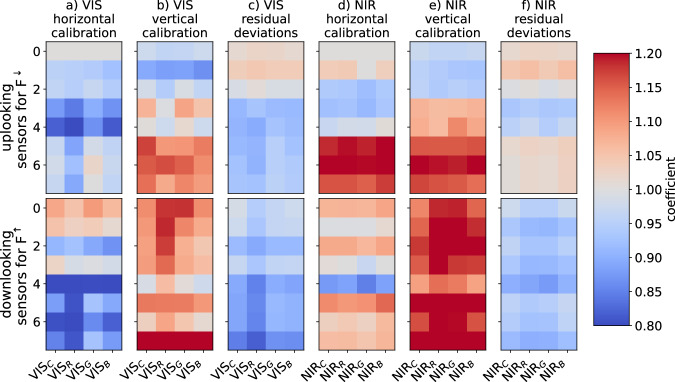
Calibration coefficients *γ*_*s,b,d/u*_ for all lightharp sensors, up-, and downward-orientated VIS and NIR and each color band, derived from the horizontal and the vertical calibration discussed in the text. Also shown is the residual derivation after the applied calibration.

Both methods yielded different correction factors shown in Fig. 6. Similarity patterns, however, were recognizable. We thus decided to retrieve the correction factor for each sensor from the average of both calibration methods. We are confident this is the best approximation to the correction factor we can obtain retrospectively. Mean absolute deviations in the vertical calibration setup after applying these factors reduce to 6% in average across all sensors with a maximum deviation of 18%.

#### Absolute calibration

**Fig. 7:**
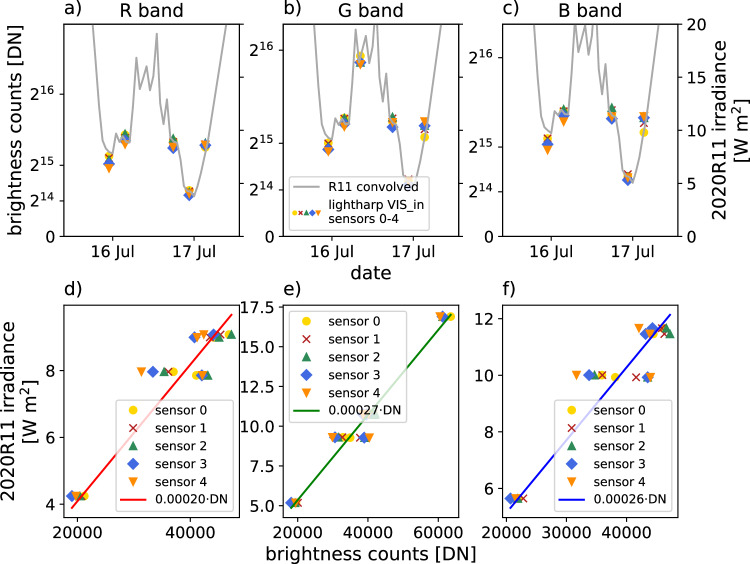
Absolute calibration of the lightharp based on irradiance measurements of radiation station 2020R11. Panels (**a**–**c**) show the time series of planar irradiance measured by 2020R11 and converted to the spectral bands R, G, and B (grey lines) and the non-oversaturated measurements of the exposed lightharp sensors 0 to 4 (markers). Panels (**d**–**f**) contain scatter plots of the lightharp measurements compared to the 2020R11 reference and the resulting linear fit function.

For the absolute calibration of the sensors to obtain irradiance measurements, we compared lightharp measurements with calibrated measurements of the radiation station buoy 2020R11^12^. The buoy was located in the vicinity of the lightharp on MOSAiC and, among others, had a TriOS RAMSES VIS ACC spectroradiometer installed, measuring wavelength-dependent planar irradiance of solar radiation above the snow from 320 nm to 950 nm with a spectral resolution of about 3.3 nm in 1 nm sampling width. On 2020-07-16 and 2020-07-17, surface melt had already exposed the uppermost 5 sensors of the lightharp to the free air. Illumination conditions on both days were completely diffuse, visually confirmed with the 360° PANOMAX panorama camera shots from RV *Polarstern*’s crowsnest^20^, and therefore non-directional and weak enough to remain within the dynamic range of the lightharp VIS color bands. The NIR sensors and the VIS_*C*_ channel were, however, oversaturated under these conditions due to the lower filtering of the incident light. It was therefore not possible to determine an absolute calibration for them, which however does not prevent the retrieval of optical properties in the NIR and VIS_*C*_ channel that only require a relative calibration. As reference data for irradiance, we convolved the 2020R11 spectroradiometer data with the relative response functions of the individual lightharp color bands, eventually yielding calibration coefficients *τ*_*b*_ to convert lightharp intensity measurements *I*_*s,b,d/u*_ into calibrated values of irradiance ${E}_{{d}_{s,b}}^{{\prime} }={\tau }_{b}\cdot {I}_{s,b,d}$ and ${E}_{{u}_{s,b}}^{{\prime} }={\tau }_{b}\cdot {I}_{s,b,u}$ (Fig. 7). The resulting calibration coefficients are listed in Table 2. Given that the relative spectral response functions are arbitrarily scaled, we note that the obtained irradiance ${E}_{d}^{{\prime} }$ needs to be scaled to provide total irradiance *E*_*d*_. One possibility is the spectral combination of PAR presented in the next chapter. The included RAMSES sensor was compared to all other RAMSES used on MOSAiC before deployment at AWI in 2019 and deviations between individual sensors were corrected during data processing.

**Table 2:** Retrieved calibration coefficients for sensor data.

Coefficient	*VIS*_*R*_	*VIS*_*G*_	*VIS*_*B*_	*VIS*_*C*_
	lightharp:			
*τ*	2.045 × 10^−4^	2.677 × 10^−4^	2.569 × 10^−4^	—
*β*	1.065 × 10^−3^	1.233 × 10^−3^	1.081 × 10^−3^	—
*α*	1.0989	0.989	1.573	—
	lightchain 2020R11 and 2020R21:			
*τ*	2.385 × 10^−5^	3.194 × 10^−5^	3.096 × 10^−5^	3.600 × 10^−5^
*β*	1.267 × 10^−4^	1.475 × 10^−4^	1.322 × 10^−4^	1.570 × 10^−4^

*τ* converts lightharp and lightchain intensity measurements into calibrated values of irradiance, *β* converts sensor measurements to photon flux density measurements, and *α* defines the linear spectral combination of lightharp measurements to PAR.

### Lightchain calibration

**Fig. 8:**
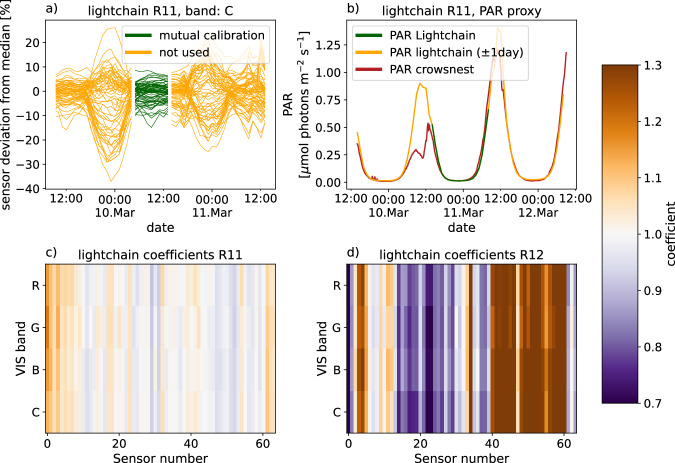
Calibration of the lightchains 2020R11 and 2020R12. (**a**) shows the variability of all sensors on lightchain 2020R11, derived as deviation from the median and the selected calibration timespan (green). (**b**) shows the absolute calibration of lightchain 2020R11 by comparing derived PAR with PAR derived from the reference RAMSES sensor. (**c,d**) include the calibration coefficients for lightchain 2020R11 and 2020R12.

The lightchains were calibrated onboard RV *Polarstern* in the days before deployment. Lightchains were horizontally mounted outside on rails for several days, with the sensors facing upwards. A TriOS RAMSES VIS ACC spectroradiometer mounted on the RV *Polarstern* crowsnest was used as a reference sensor for absolute calibration. We manually selected timespans to calculate correction factors for differences between sensors (Fig. 8a) and timespans for absolute calibration (Fig. 8b). Both calibrations used the same retrieval methods as for the lightharp. However, for sensor inter-calibration, we used the median intensity of all sensors as a reference instead of the uppermost sensor, resulting in calibration coefficients for the 2020R11 lightchain between 0.9 and 1.2 (Fig. 8c). The mounting of the 2020R12 lightchain was probably not leveled enough, or it was contaminated with artificial light sources, resulting in comparably large coefficients (Fig. 8d) that increased the spikiness of measurement profiles below the ice, where we would expect a steady light field. Hence, we omitted the calibration of the 2020R12 sensor. Absolute calibration coefficients *τ*_*b*_ are given in Table 2. Since the reference sensor in the crowsnest was not regularly maintained at the time of calibration, and could therefore have been covered by ice crystals, for example, the absolute values of the lightchains should only be used with caution.

### Deployment information and auxiliary data

Lightharp and lightchains were deployed on MOSAiC at study sites dedicated to investigating light transmission, ice mass balance, and sea ice physical and biological properties. These sites and their drift trajectories through the Arctic Ocean are described in Nicolaus *et al*.^9^. The instruments were directly co-located with or part of radiation stations and ice mass balance buoys. Instruments were deployed in boreholes in the ice, which refroze directly afterward. Drilled holes were only slightly bigger than the instrument’s radius to minimize the deployment impact of the subsequently non-destructively measuring devices.

#### Lightharp

**Fig. 9:**
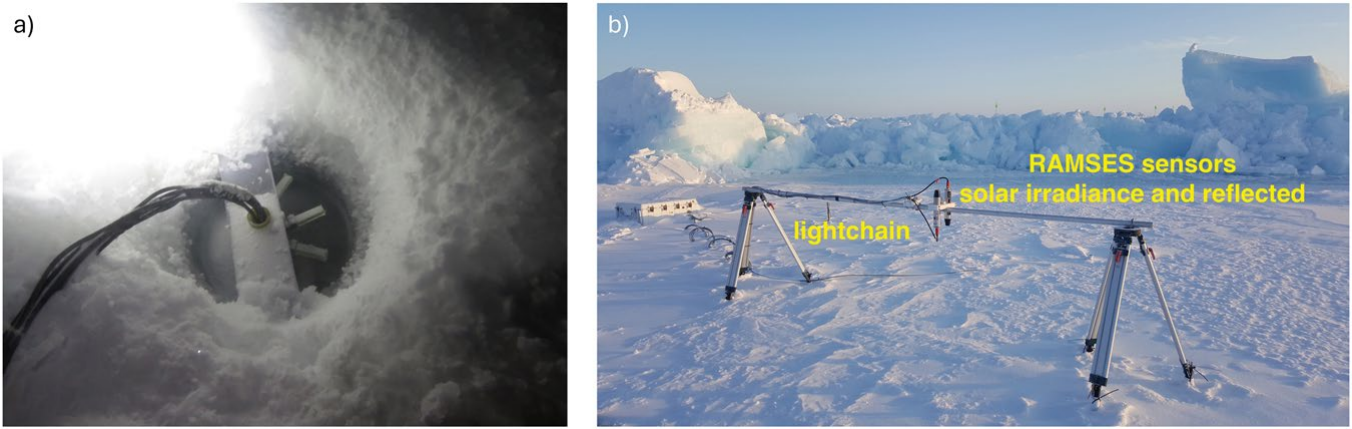
Pictures of deployed system. (**a**) Lightharp just deployed into the ice. (**b**) Radiation station on MOSAiC with RAMSES spectroradiometers measuring solar incident and reflected irradiance. Lightchain peeks out of the snow in the background.

The lightharp system was deployed in direct vicinity to the second-year ice dark coring site (MOSAiC *LM site*) at 87°28.6′N, 103°12.4′E on 2020-01-15 (Device Operation *PS122/2_14-314 lightharp_1*), a month before the first nautical twilight conditions after the end of the polar night (sun elevation above 12° below the horizon). The deployment hole (Fig. 9a), drilled with an 8-inch ice auger entirely through the ice, was completely cleaned from slush but started to refreeze immediately in the conditions of −26.5 °C air temperature and 10 ms^−1^ wind with moderate snow drift. It took about one week until the drill hole was refrozen down to the lowest sensor, as indicated by temperature sensor readings. Seawater temperature was around −2.0 °C and practical salinity 28.7 during deployment measured with handheld devices, so these are only approximations of the real values which explains the apparent oceanic temperature below freezing. The uppermost sensor module was aligned precisely with the ice surface, 0.07 m above the water level, matching the measured freeboard height (0.07 m) of the 1.18 m thick ice with a snow cover of 0.17 m. All initial sensor depths were thus as previously specified reaching from 0.0 m to 1.4 m, measured from the snow–ice interface downwards. They were valid until the onset of sea ice surface melt on 2020-06-18, detected by the matching daily temperature trend at the top sensor module (module 0) with the air temperature recorded by buoy 2019T62^21^.

The recording frequency of the instrument was set to one profile every 5 h to cycle through different daytimes, unaffected by the drift through many different longitudes and, therefore, various times of solar peak close to the North Pole.

The system was co-deployed with various other sensors at the *LM site* as listed in Table 3.

**Table 3:** Overview on co-deployed sensor buoys at the MOSAiC *LM site*.

Buoy ID	Type	Deployment	Data source
2020M29	IMBflex buoy	Leg 2	—
2020R11	Radiation station	Leg 3	DOI
2019S96	Snow buoy	Leg 1	DOI
2019T62	SIMBA buoy	Leg 1	DOI
2019T66	SIMBA buoy	Leg 1	DOI
2020E3	OptiCAL ‘hh’/EnviPOPE	Leg 2	DOI
saltharp_1	Saltharp	Leg 2	DOI
lightharp_1	Lightharp	Leg 2	DOI

#### Lightchains

Lightchains were deployed as part of the radiation stations 2020R10^15^, 2020R11^12^, 2020R12^14^, and 2020R21^16^ (Fig. 9b). 2020R11 at the *LM site* was deployed at 85°58.8′N, 13°5.4′E on 2020-03-26 and measured in the ice until 2020-07-22. The deployment depth of the uppermost sensor 1 was 0.15 m above the ice surface. Given the 0.05 m vertical spacing, sensor 4 was exactly at the snow–ice interface. One sensor string of the system failed, leading to a data gap between 1.45 m to 1.8 m depth. Ice thickness during deployment was 1.63 m, with 0.23 m snow layer above and an ice freeboard of 0.20 m. The lightchain as part of system 2020R12 was deployed on the *L3 site* in the Distributed Network at 83;55.6 N, 14;45.7E on 2020-4-24 in 1.67 m thick first-year ice, with 0.08 m snow layer on top and 0.26 m freeboard. It measured until 2020-08-07. Sensor 3 was at the initial snow–ice interface. Radiation station 2020R11 and its lightchain were redeployed at 87;57.0 N, 107.36E on 2020-08-27 as radiation station 2020R21 on MOSAiC leg 5 within a pond that froze shortly after. Ice thickness of the pond bottom was 0.82 m and pond depth 0.31 m. The pond–ice interface was located between sensors 15 and 16. The lightchain of system 2020R10 located in the *Central Observatory* was unfortunately strongly affected by malfunctions, starting two weeks after deployment. So we exclude it here from additional analysis and just included the profiles measured successfully directly after deployment on 2020-03-08. It got deployed on level FYI between the end of the ROV optics grid (Transponder 2) and Fort Ridge in 1.49 m thick ice with 0.28 m freeboard and 0.07 m of snow on top. It measured until 2020-03-23. In summer, bio-fouling or algal growth occurred on the lightchain sensors exposed to the water column, leading to an identifiable spectral change.

### Derived quantities

#### Scalar irradiance

The implementation of sensors measuring planar irradiance allows us to quantify the radiant energy flux through horizontal planes, an essential quantity for physical processes (^10^, and references therein). In contrast, scalar irradiance quantifies the total radiant energy entering a test volume from all directions equally and is crucial for biological applications. To convert the planar irradiance measured by the lightharp sensors to spherical scalar irradiance, we add down- and upwelling planar irradiance and multiply it by 2, assuming a perfectly isotropic light field. Katlein and colleagues^10^ found that this is a good proxy for converting irradiance at the sideward-oriented planar sensors of the lightchain to hemispheric scalar irradiance. We note that Katlein and colleagues^22^ found with Monte Carlo simulations a conversion factor of about 1.6 for downwelling light below sea ice, caused by a downward directed light field in the ice, resulting from an anisotropic scattering function of the ice. Comparable measurements or simulations are missing for the light field in the ice, especially above the asymptotic regime, and for upwelling light. Hence, we neglect any higher complexity here. Lightchain values are converted from sideward planar to spherical scalar irradiance by multiplication with the factor 4^10^.

#### PAR irradiance

To better understand the response of the ecosystem to changing light conditions, it is necessary to quantify how many photons enter the ice in PAR wavelengths that stimulate primary productivity (400 nm to 700 nm). PAR is quantified by the photosynthetic photon flux density (PPFD). The broad PAR wavelength range includes more accurate response functions of the different chlorophyll types. To retrieve the PPFD from lightharp measurements, we first converted the 2020R11 irradiance ${E}_{{d}_{\lambda }}$ measurements to spectral-dependent photon flux density ${E}_{{d}_{\lambda ,phot}}$ using

10 $$k(\lambda )=\frac{\lambda }{hc\cdot {N}_{A}}=\frac{\lambda [nm]}{1.196\cdot 1{0}^{2}}\frac{\mu mol\;photons}{J}$$

11 $${E}_{{d}_{\lambda ,phot}}=k(\lambda )\cdot {E}_{{d}_{\lambda }}$$

with Planck’s constant *h*, speed of light *c* and Avogadro number *N*_*A*_.

After convolving the spectra with the relative spectral response functions of the implemented sensors, we calibrated all three VIS color bands *b* of the lightharp to these reference values to retrieve photon flux density measurements ${E}_{{d}_{s,b,phot}}^{{\prime} }={\beta }_{b}\cdot {I}_{s,b}$, yielding the band-specific calibration coefficients *β*_*b*_ listed in Table 2. As a last step, we optimize a linear combination of the RSR functions of the VIS color bands to match the PAR response function:

$$PA{R}_{phot}=\left\{\begin{array}{ll}1 & 300\,{\rm{nm}}\le \lambda \le 700\,{\rm{nm}}\\ 0 & {\rm{else}}\end{array}\right.,{\rm{for}}\;{\rm{wavelengths}}\;\lambda .$$

Table 2 contains the coefficients *α*_*b*_ of the linear combination to retrieve the proxy for PAR irradiance:

12 $$PA{R}_{phot}=\left({\alpha }_{R},{\alpha }_{G},{\alpha }_{B}\right)\left[\begin{array}{c}{E}_{{d}_{s,R,phot}}^{{\prime} }\\ {E}_{{d}_{s,G,phot}}^{{\prime} }\\ {E}_{{d}_{s,B,phot}}^{{\prime} }\end{array}\right]$$

with _*phot*_ indicating that the irradiance is given in photon flux density as previously calibrated. We combined only the three VIS color channels R, G, and B to PAR, as we had no absolute calibration of the NIR sensor due to oversaturation. The lightchain was calibrated similarly, using spectroradiometer data measured on RV *Polarstern* during the calibration.

**Fig. 10:**
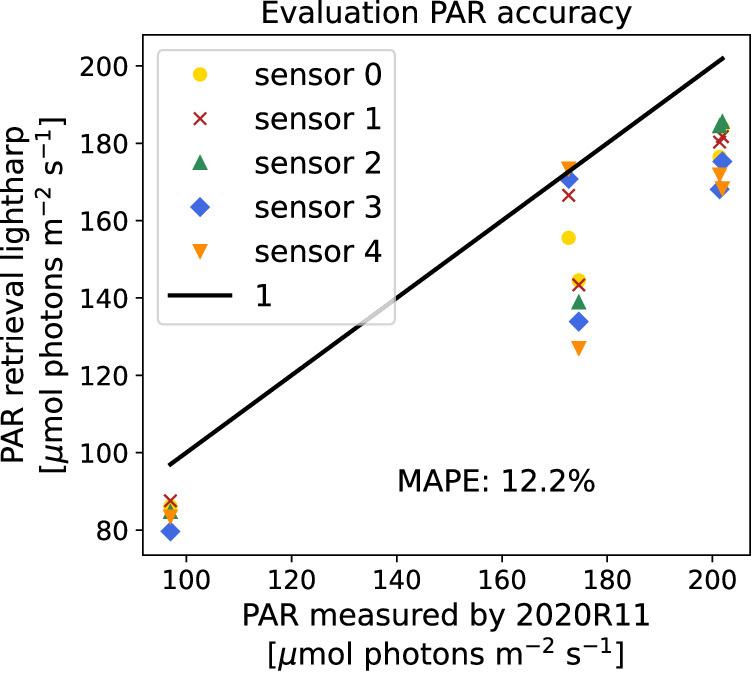
PAR retrieved from the lightharp in free air in comparison to PAR measured by radiation station 2020R11^12^.

To test the retrieval method, we compared the lightharp PAR proxy to PAR measurements from 2020R11^12^, derived from a conversion of the data to photon flux density and subsequently integrated over the PAR wavelength range (Fig. 10). Results show that the planar incident PPFD of the lightharp is slightly underestimated with a MAPE of 12.2% during the calibration days 2020-07-16 and 2020-07-17 at which the sensors were exposed to free air. This underestimation of PPFD compared to the reference sensor can be almost entirely attributed to the low-pass filter cutoff at approximately 650 nm on the VIS sensor module implemented on the lightharp and lightchains. This is confirmed by a comparison of PPFD measured by the radiation station 2020R11^12^ in the full PAR spectrum and, for comparison, in the linear combined PAR spectrum from the R, G, and B color bands, revealing an underestimation by 11.7% caused by the more narrow spectra. Since snow and ice are optically thick for long wavelengths, we assume the error will diminish within the ice and below and, therefore, do not further correct this deviation.

**Fig. 11:**
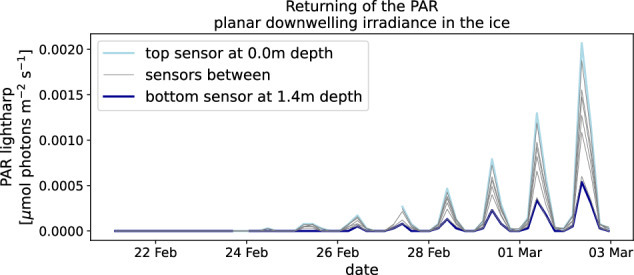
Downwelling planar PAR irradiance measured with the lightharp in the ice on MOSAiC at the end of the polar night.

In the highest gain factor, the lightharp measured a planar PAR irradiance not higher than 2.54 × 10^−5^ μmol photons s^−1^m^−2^ on 2020-02-24, with a steady incline on the days afterward around noon time. The complete measurement range reached from 0 to about 254 μmol photons s^−1^m^−2^. Resolving the daily cycle of incoming irradiance during the light return after polar night without any fluctuations other than the daily cycle (Fig. 11), the lightharp proves that dark or background noise is sufficiently suppressed in the acquisition electronics and the given resolution at the highest gain factor equals the minimum detectable signal. The first PAR signal on 2020-02-24 was detected, when the sun elevation was still 8.4° below the horizon. This underlines the sensitivity of the built-in sensors to detect photons even in nautical twilight under a 20 cm snow cover.

#### Derivation of reflectance, transmittance, absorptance, and slab albedo

Apparent optical properties of the ice layers between the lightharp sensor modules are derived following the layer model described in section *Layer scheme* by equating the conceptually introduced vertical fluxes *F*_↑_ and *F*_↓_ to the measured planar irradiances *E*_*u*_ and *E*_*d*_. Furthermore, we derive a slab albedo from the ratio of up- to downwelling irradiance at each sensor module. This slab albedo, in standard terminology: irradiance ratio^23^, equals the ice albedo as if all layers above the modules were removed. The slab albedo is a purely informative value as it neglects the impact of overlying ice layers on the directional and spectral composition of the downwelling light.

#### Diffuse attenuation coefficients

Diffuse attenuation coefficients are AOPs used to quantify the reduction in irradiance of a mainly diffuse light field passing through a medium. We derive it here as a simple yet valuable approximation of the vertical light attenuation in the ice. The diffuse attenuation coefficient is derived between adjacent sensor pairs *s*_*i*_ and *s*_i+1_ in depth *d* as a scalar variable *κ* for the lightchain and lightharp, and as directional variables *κ*_*u*_ and *κ*_*d*_ for the up- and downwelling measurements of the lightharp using the scattering-free, simplified form of the radiation transfer equation:

13 $$\kappa =-ln\left(\frac{{I}_{{s}_{i+1}}}{{I}_{{s}_{i}}}\right)/(d({s}_{i+1})-d({s}_{i})).$$

#### Vertical sensor position

The relative vertical position of the sensor modules within the ice changes whenever the snow cover and the sea ice itself change their thickness through growth and melt. During deployment, we measured the initial sensor depths, which describe the vertical position during the freezing season, measured downwards positive from the interface of solid ice to snow. However, with the onset of surface melt, sensor positions in the ice start to move relative to the surface. To account for this, we derived an additional, time-dependent, vertical coordinate for the sensors that we refer to as the *ice depth*. For the lightharp, this can best be derived from the measured daily fluctuations of *in situ* temperature, as these are much higher in free air than within snow or ice. We also compare these readings to adjacent air temperature sensors, which then allow us to reliably estimate the time when a particular module had melted free from the ice and was exposed to direct solar irradiance. We use these times as anchor points for a reconstructed surface melt function. Surface melt data of an auxiliary sensor (SIMBA-type ice mass balance buoy 2019T62 at the *LM site*^24^) was incorporated to describe the melt rate between these points. For the lightchains, the ice depth coordinate was derived solely from the surface melt data of co-deployed ice mass balance buoys. While the original vertical sensor positions, which remained constant until melting, were measured with centimeter precision, the surface melting corrected positions depend heavily on inaccuracies in the reference products used and spatial differences. We, therefore, do not expect an accuracy better than one vertical sensor distance, which is 5 for the lightchains and for the lightharp a minimum of 1.5 and gradually more with the increasing sensor distances downwards (validated with the reconstructed surface melt function).

#### Snow and ice thickness

At both sites (LM and L3), where lightharp and lightchain (2020R11), respectively (2020R12) measured over a long period of time, derived snow and ice thickness values are available from co-deployed SIMBA buoys^24^. For each site, we determined statistical values of mean, max, min and standard deviation of both physical properties. Buoy 2019T62 was particularly representative for the lightharp and, therefore, added separately to the dataset.

### Data exclusion

As the lightharp and lightchains are not under permanent surveillance, an integrity check of the data was performed to detect and exclude suspicious measurements. These included:

- times after the entire lightharp had probably tipped due to excessive surface melt
- lightchain measurements as part of radiation station 2020R10 (installed in the *Central Observatory*) after 2020-03-23 because of a system failure two weeks after deployment.

For other specific cases, the data should only be used with particular care or be excluded altogether, depending on application:

- All measurements with a negative ice depth coordinate were recorded in free air (lightharp) or free air and snow (lightchain) and should not be included in studies of in-ice optical properties.
- Direct radiation in clear sky conditions causes a daily cycle in the measurement data. As discussed later in section *Technical evaluation and limitation*, this was caused not only by ice optical properties but also by some unavoidable mutual shading of the sensors.

## Data Record

The derived lightharp and lightchain measurement record from MOSAiC is available under 10.1594/PANGAEA.963743^25^, and includes the data arrays listed in Table 4. The data were compiled by time and deployment depth in a NetCDF file following the CF conventions^26^ for a *profile trajectory*. All retrievals and auxiliary data are presented in section *Derived quantities*. For each trajectory, the timestamp of the uppermost available measurement was adapted as the measurement time of the entire profile. We call this the *nominal date* according to the nominal spatial coordinate implemented in CF conventions.

**Table 4:** Lightharp and lightchain data product, available for (*x*) or based on (*o*) the marked spectral bands, given as interface property at the specific depth (*i*) or layer property (*l*) of the layer reaching from the interface depth *i* to *i* + 1.

Quantity	Unit	Variable name	VIS_*R, g, B*_	VIS_*C*_	NIR_*R, g, B*_	NIR_*C*_
lightharp						
I_*d*_	DN	[VIS,NIR]_in_[R,G,B,C]	x,i	x,i	x,i	x,i
I_*u*_	DN	[VIS,NIR]_out_[R,G,B,C]	x,i	x,i	x,i	x,i
I_*o*_	DN	[VIS,NIR]_scalar_[R,G,B,C]	x,i	x,i	x,i	x,i
PAR planar ↓	μmol photons s^−1^m^−2^	PAR_proxy_in	o,i	—	—	—
PAR planar ↑	μmol photons s^−1^m^−2^	PAR_proxy_out	o,i	—	—	—
PAR scalar	μmol photons s^−1^m^−2^	PAR_proxy_scalar	o,i	—	—	—
transmittance	1	transmissivity	x,l	x,l	x,l	x,l
reflectance	1	reflectivity	x,l	x,l	x,l	x,l
absorptance	1	absorptivity	x,l	x,l	x,l	x,l
slab albedo	1	slab_albedo	x,i	x,i	x,i	x,i
planar attenuation downwelling *κ*_*d*_	m^−1^	[VIS,NIR]_downwelling_attenuation_[R,G,B,C]	x,l	x,l	x,l	x,l
planar attenuation upwelling *κ*_*u*_	m^−1^	[VIS,NIR]_upwelling_attenuation_[R,G,B,C]	x,l	x,l	x,l	x,l
scalar attenuation	m^−1^	[VIS,NIR]_scalar_attenuation_[R,G,B,C]	x,l	x,l	x,l	x,l
temperature	°C	T_deg	i			
lightchains						
I_→_	DN	VIS_side_[R,G,B,C]	x,i	x,i	—	—
scalar attenuation	m^−1^	VIS_scalar_attenuation_[R,G,B,C]	x,i	x,i	—	—
auxiliary data (if available for site and time)						
ice depth	m	ice_depth	corrected vertical sensor position			
snow thickness	m	snow_thickness	mean, max, min and standard deviation			
ice thickness	m	ice_thickness	mean, max, min and standard deviation			
location	°	lat, lon	geographical position in WGS-84			

Most variables are provided uncorrected and corrected (suffix *_cal* in the variable name) for mutual brightness differences derived from the calibration. Auxiliary data were added when collocated autonomous buoys were available, from which the data could be derived.

The data of the lightharp are split into interface properties measured at the sensor depths and layer properties determined for the layers between the sensor modules. Layer properties of the lightharp are averaged between sensors 1 and 3 because of an irradiance increase from sensor 1 to 2 within the highly scattering regime, making the retrieval of layer apparent optical properties from the system ambiguous. Such light increase has been observed before with the lightchain^10^. The lightharp system measured between 2020-01-15 and 2020-08-13, with only a few isolated missing data points and with interpretable data until 2020-07-18T07:41:42 UTC. After that, the system probably tipped over. At the end of leg 4, when the floe on which the instrument was installed drifted away from the main study floe, the lightharp could unfortunately not be recovered. As described above, this prevented a post-deployment calibration and a final read-out of the memory card. On 2020-08-13, the system likely sank in Fram Strait, as data transmission stopped then.

We retrieved a proxy for surface melt at the lightharp and lightchains locations, and calculated the time-dependent ice-depth array in addition to the deployment depths. Sensor and ice depths specify the depth of the layer interfaces. Values measured at these interfaces are marked with *i* in Table 4. Layer properties marked with *l* are given between two interfaces, defined by the coordinates marked with *top* and *bottom* in the files. All 16-bit DN data points equalling 65535 were replaced by *Nan* to avoid misinterpretation caused by oversaturation.

Additional light measurements were obtained, for example, by the AWI ROV *Beast* that measured the horizontal distribution of transmitted irradiance at regular time intervals^27^, by manually operated L-Arms used for an event-based determination of transmitted irradiance, by PAR sensors mounted on the CTD and ITPs^8^, and by autonomous ice-tethered light profilers (OptiCALs) that measured planar irradiance in different depths below the ice^28–30^.

## Technical Validation

Besides a manual in-depth review of individual data points, we tested the entire data set for validity and, since the lightharp system had never been evaluated to such an extent before, also for systematic shortcomings and limitations.

### General evaluation

**Fig. 12:**
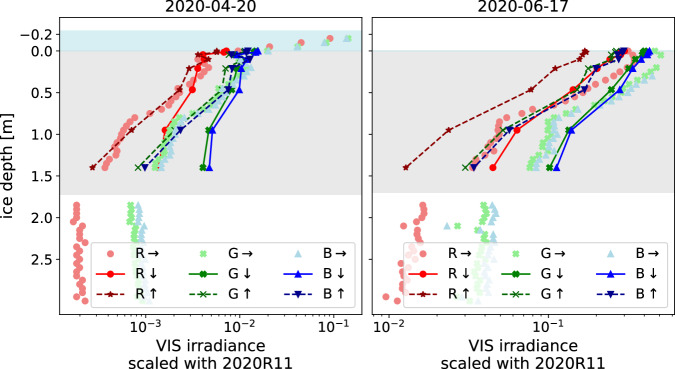
In-ice light intensity profiles at the *LM site* on two exemplary days measured with the lightharp (downward planar $\mathop{d}\limits_{ \sim }\downarrow$, upward planar $\mathop{u}\limits_{ \sim }\uparrow$) and lightchain 2020R11 (sideward planar →). Shown are measurements with the three VIS color bands R, G, and B, scaled with the downwelling planar intensity at radiation station 2020R11^12^. The grey box shows the vertical extent of the ice, and the blue box shows the extent of the snow layer retrieved from 2019T62^24^.

Vertically resolving, non-destructive light measurements from within sea ice are scarce. Therefore, only the data published in Katlein *et al*.^10^ were available to assess the general validity of our data relative to previous measurements. We selected two example profiles from 2020-04-20 (snow-covered) and 2020-06-17 (snow almost entirely melted) to compare distinct features of the in-ice light field under complete diffuse illumination conditions on both days. Measured planar irradiance was scaled with the downwelling planar irradiance measured by 2020R11^12^ above the snow cover in ≈1 m height, extracted with the color band relative spectral responses. Figure 12 shows that the lightharp and lightchain reproduced important features that also Katlein and colleagues (2021) observed in their study. This includes, from a purely qualitative perspective:

- exponential light decline in the ice from top to bottom
- weaker back reflection close to the ice bottom in April due to a downward photon loss at the ice–ocean interface, which was at that time about 0.3 m below the lowest lightharp sensor
- slight downward light increase in a depth of 0.1 m to 0.2 m (lightharp) and 0.2 m to 0.3 m (lightchain 2020R11) on 2020-04-20, probably due to local scattering effects (observed in the reference study^10^ down to a depth of ≈0.5 m)
- stronger absorbance of wavelengths in the red spectrum in comparison to blue and green, especially in snow
- much stronger light attenuation within the snow cover in comparison to sea ice

Additionally, the calibration method with an *in-situ* reference system allows for the reliable evaluation of the measured absolute values.

### Sensitivity to the angle of incident light

**Fig. 13:**
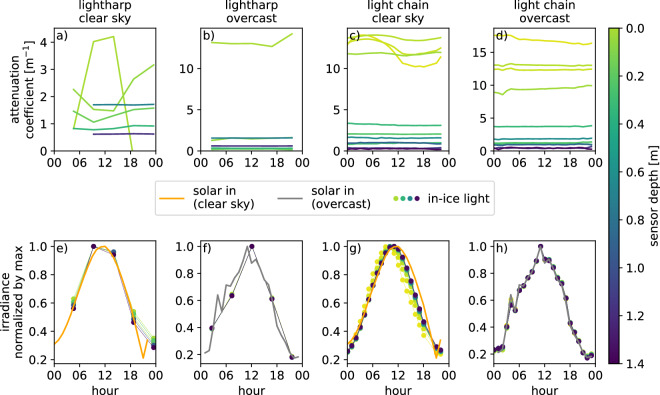
Impact of the daily solar irradiance cycle on the in-ice measurements separated by lightharp (**a**,**b**,**e**,**f**) and lightchain (**c**,**d**,**g**,**h**) and solar incident light conditions: diffuse solar irradiance (2020-04-15) (**b**,**d**,**f**,**h**) and clear sky irradiance (2020-04-23) (**a**,**c**,**e**,**g**). Panels (a-d) show the diffuse attenuation coefficients in different ice depths. Panels (**e**–**h**) show the daily solar irradiance cycle above the snow and ice and in different ice depths scaled with the daily maximum.

In order to examine the impact of the angle-dependent exposure of sensor modules to partially directional solar radiation in the upper layers of the ice, we compared the data from days with clear sky conditions to data from overcast days with their diffuse solar irradiance. We compare lightharp and lightchain data here, as their deviating sensor alignments, helically aligned (lightharp) or all oriented in the same direction (lightchain), make them particularly suitable for comparison, indicating which factors play a significant role. Results show that measured irradiance in different depths in the ice mainly resembles the daily cycle of solar irradiance in free air (Fig. 13e–h). We find a slightly reduced daily amplitude of irradiance at the uppermost lightharp sensors under clear sky conditions in comparison to the sensors deeper in the ice (Fig. 13e). This vertical amplitude difference does not occur with the lightchains in clear sky. However, the daily maximum at the upper sensors is shifted by 2 h earlier and to about 1 h earlier from sensor 5 downwards (Fig. 13g). In overcast conditions, all measurements go along with the daily cycle (Fig. 13f,h). For the lightharp, trends are more difficult to compare due to the limited temporal resolution of 5 h.

Together, these observations indicate that the sideward-looking sensors of the lightchain are slightly affected by the solar azimuth, causing a measured -1 h phase shift of the daily cycle relative to the actual daily cycle. The slight underestimation of the amplitude of the daily cycle by the upper lightharp sensors is likely caused by elevation-dependent penetration of directional light into the ice.

For a more in-depth analysis of the measurements, we investigated the derived attenuation coefficients that quantify the vertical decrease of light in the ice. These parameters, resolving small-scale differences between layers, seem to be somewhat unreliable under clear sky conditions in the upper ice layers (Fig. 13a,c). Under these conditions, they show clear sub-daily fluctuations whose phase is not necessary aligned with the sun elevation and unevenly shifted at the different depths (Fig. 13a). This indicates that the azimuth angle of solar radiation also impacts the lightharp sensor measurement. Supporting this hypothesis, the phase shift on the lightchain with constantly aligned sensors is largely depth invariant (Fig. 13c). At the same time, the phase differs up to 12 h on the lightharp with helical alignment, probably caused by the shading of sensors. Therefore, we conclude that the estimation of light attenuation coefficients under clear sky conditions is possibly erroneous at depths above 0.4 m at the lightharp and at depths above 0.2 m at the lightchains. In diffuse, overcast situations, this error does not occur (Fig. 13b,d).

### Negative absorptance in lightharp AOP retrievals

**Fig. 14:**
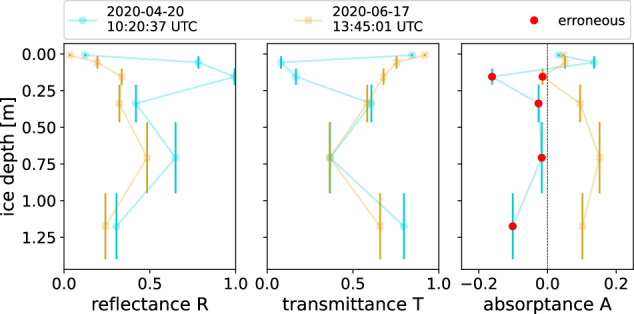
Profiles of apparent optical properties reflectance, transmittance, and absorptance derived for in-ice layers between the lightharp sensor modules on 2020-04-20 and 2020-06-17. Red dots mark negative absorptance.

We observed negative values in the derived absorptance in layer 3 (0.1 m to 0.21 m ice depth) and temporarily also in the underlying layers 4, 5, and 6 (Fig. 14). In the lowest observed layer 6, absorptance increased steadily when the ice grew thicker below.

**Fig. 15:**
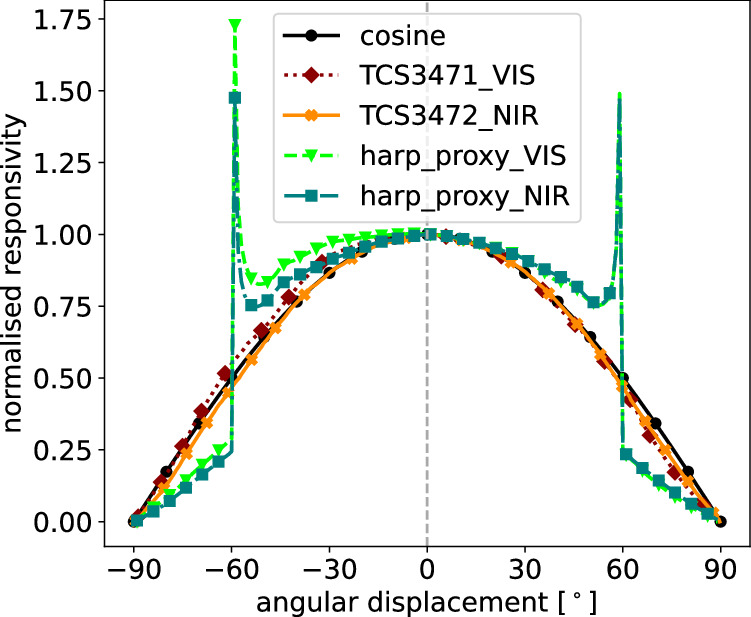
Theoretically obtained responsivity of the molded lightharp sensor modules for different elevation angles of incident light.

Having tested other sources of error, like reflection within the molded sensor modules or directional solar radiation on clear-sky days, we conclude that the effect is probably from the anisotropic light field in the ice^22^. Hence, the energy-conserving layer model (Eq. 6) cannot be transferred unconditionally to the lightharp because the sensors do not fully resolve the net radiation. Figure 15 shows a theoretical approximation of the angular responsiveness of the sensor modules based on the manufacture specifications of the sensors and theoretically approximated optical properties of the resin molding. The molding changes the cosine-like angular dependence of the sensors so that they further lose responsiveness to lateral radiation. We considered refraction at the ice–resin interface, attenuation in the molding, and the finger-like shape of the modules. At 59°, responsiveness peaks resulting from light beam bundling caused by refraction. Being less responsive for lateral incident light, the lightharp probably underestimates back reflected light entering the layer from below when the angular distribution of the upwelling light *F*_↑_ changes from a layer top to bottom to more horizontal beam directions.

We conclude that the negative values in absorptance result from an incomplete understanding of the in-ice light field, which shows a depth-variant angular radiance distribution (as recently shown by Larouche *et al*.^31^), presumably amplified in the reflected light field that, together with the lightharp sensor optics, causes slight deviations. This deviation, however, has only a minor effect on the lightharp measurements, since the behavior is limited to a small portion of the total data set and only affects absolute AOPs, but not how they change relative to each other and over time.

### Concluding limitations

In the technical validation, we found that the newly developed lightharp system is sensitive to the solar azimuth angle in clear sky conditions. Furthermore, we found that the angular distribution of radiance probably changed with depth, and does not reach an isotropic regime throughout the ice body, which causes minor uncertainties in the apparent optical properties derived from lightharp data. To reduce the influence of these constraints and of the relatively long measurement cycles of lightharp profiles on the derived optical properties, we recommend averaging from multiple profiles from several consecutive days. The lightchains were slightly impacted by the sun azimuth, resulting in a phase shift of the daily amplitude and a weak diurnal cycle in derived attenuation coefficients in the uppermost ice layer.

In general, optical measurements in and below sea ice can be affected by small-scale variations in the optical light field^32^ caused by variations in snow and ice thickness, surface meltwater and flooding, ice structure and any kind of inclusions such as sediments, algae and gas bubbles. Furthermore, it is possible that the refreezing process of the ice in the deployment hole and around the instrument body influences the results, even if we assume that, as with other measurements of physical properties, this effect is small in natural conditions of top-down ice growth. Laboratory tests with comparable sensor designs^33^ showed that the same conditions for brine dynamics as for the earlier-grown ice led to a similar ice structure around the sensor after refreezing. Optical sensors are also less affected due to their large field of view, and the deployment of the instruments early in the year ensured that the ice had enough time to smoothen out small variations before the return of significant solar radiation in summer.

## Usage Notes

Raw and processed lightharp and lightchain data from the MOSAiC expedition are available on PANGAEA^25^, under the license CC-BY-4.0. We suggest using the *Python Xarray* package^34^ to work with the NetCDF data. Alternatively, all standard programs complying with CF conventions^26^ should be able to read and work with the data, as these have been implemented as far as they were defined for in-sea ice data.

## Data Availability

Data processing can be retraced in the GitHub project https://github.com/nielsfuchs/MOSAiC_inicelight/. Files containing the derived calibration coefficients for the lightchains and the lightharp are included into the repository.
